# Timing, Frequency and Environmental Conditions Associated with Mainstem–Tributary Movement by a Lowland River Fish, Golden Perch (*Macquaria ambigua*)

**DOI:** 10.1371/journal.pone.0096044

**Published:** 2014-05-02

**Authors:** Wayne M. Koster, David R. Dawson, Damien J. O’Mahony, Paul D. Moloney, David A. Crook

**Affiliations:** 1 Arthur Rylah Institute for Environmental Research, Department of Environment and Primary Industries, Heidelberg, Victoria, Australia; 2 School of Life and Environmental Sciences, Deakin University, Warrnambool, Victoria, Australia; Institute of Marine Research, Norway

## Abstract

Tributary and mainstem connections represent important links for the movement of fish and other biota throughout river networks. We investigated the timing, frequency and environmental conditions associated with movements by adult golden perch (*Macquaria ambigua*) between the mainstem of the mid-Murray River and a tributary, the Goulburn River, in south-eastern Australia, using acoustic telemetry over four years (2007–2011). Fish were tagged and released in autumn 2007–2009 in the mid-Murray (*n* = 42) and lower Goulburn (*n* = 37) rivers within 3–6 km of the mid-Murray-lower Goulburn junction. 38% of tagged fish undertook mainstem–tributary movements, characterised mostly by temporary occupation followed by return of fish to the original capture river. Approximately 10% of tagged fish exhibited longer-term shifts between the mainstem and tributary. Movement of fish from the tributary into the mainstem occurred primarily during the spawning season and in some years coincided with the presence of golden perch eggs/larvae in drift samples in the mainstem. Many of the tributary-to-mainstem movements occurred during or soon after changes in flow. The movements of fish from the mainstem into the tributary were irregular and did not appear to be associated with spawning. The findings show that golden perch moved freely across the mainstem–tributary interface. This demonstrates the need to consider the spatial, behavioural and demographic interdependencies of aquatic fauna across geographic management units such as rivers.

## Introduction

In lotic ecosystems, connections between tributaries and the mainstem function as conduits for the flow of organic material and energy, and are critical for supporting riverine biodiversity and maintaining habitat heterogeneity [1]–[3]. Connections between tributaries and mainstem habitats may also act as corridors for the movement and migration of aquatic fauna by linking geographically distinct populations across the river network [4]. While many previous studies of fish movement in rivers have focused on the ecological importance of longitudinal (i.e. along a river channel) and lateral (i.e. river–floodplain) movements [5]–[8], there is growing evidence that mainstem–tributary movements are important for maintaining fish populations in river networks [9], [10]. For example, some species move between mainstem habitats and tributaries to avoid unfavourable conditions during high flows [10], [11] whilst others undertake such movements to take advantage of specific food resources [12].

Hydrological regimes are a major driver of river ecosystems and provide cues for a range of important behaviours in fishes, including movement and spawning [13]–[16]. In regulated rivers, hydrological regimes are often modified to the extent that these cues are disrupted or missing, which can lead to impacts on native fish populations such as reduced diversity and abundance [17], [18]. However, flow restoration in regulated rivers through the allocation of environmental flows provides an opportunity to deliberately deliver these behavioural cues, with the aim of restoring fish populations [19]. One of the main challenges in the science and management of environmental flows, therefore, is developing an understanding of the often spatially and temporally complex relationships between fish behavioural responses and river discharge [13], .

The aim of the current study was to examine the movements of a widely distributed native Australian fish species to determine the frequency and timing of movement between mainstem and tributary habitats in a lowland river system. The study species, golden perch *Macquaria ambigua*, is a popular recreational angling species found in lowland rivers and lakes in south-eastern Australia. Golden perch in rivers display strong site fidelity and occupy restricted ranges (usually <0.5 km) for extended periods [21]–[23], but may also move long distances (e.g. tens or even hundreds of kilometres) during increases in flow and water temperature in spring [22], [24], [25]. There is some evidence that golden perch tend to congregate at mainstem–tributary junctions during the spawning season in late spring and early summer [22]. Acoustic telemetry was used to determine whether adult golden perch move between a mainstem river and the lower reaches of a major tributary, whether the frequency and/or predominant direction of any mainstem–tributary movement change during the spawning period, and whether hydrology or temperature influences the occurrence of mainstem–tributary movements.

## Materials and Methods

### Ethics statement

This study was conducted under Victorian Flora and Fauna Guarantee Permit 10004894, Fisheries Victoria Research Permit RP-827, New South Wales Scientific Collection Permit P09/0076 and ethics permits 06/24, 07/08 and 09/14 (Arthur Rylah Institute Animal Ethics Committee).

### Study site

**Figure 1:**
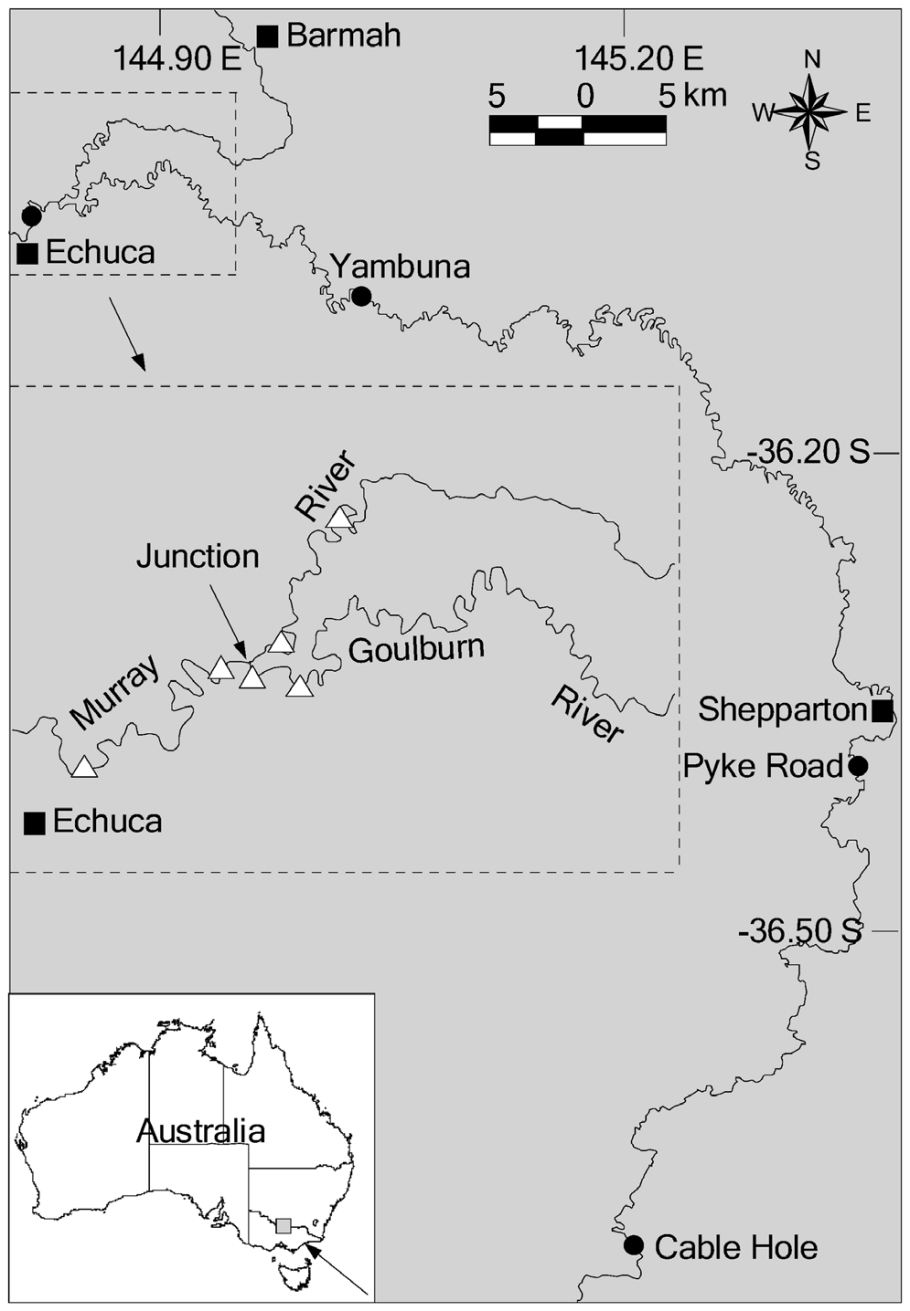
Map showing location of the study site. White triangles represent the locations of each of the listening stations in the Murray and Goulburn rivers. Black circles represent the locations of each of the drift sampling sites.

The study was conducted in the mid-Murray and lower Goulburn rivers in south-eastern Australia (Fig. 1). Much of the catchment is cleared agricultural land although some areas of forest remain, particularly in the lower reaches which flow through the Lower Goulburn National Park. Flow in the mid-Murray and lower Goulburn rivers is highly regulated by several upstream dams and weirs, which in particular have reduced winter–spring flows, and this has likely impacted on life cycles and recruitment processes of native fish (e.g. through reduced duration of freshes that serve as spawning and/or migration cues) [26], [27]. Average annual discharge in the mid-Murray and lower Goulburn rivers is about 4 661 000 and 1 340 000 ML, respectively [28], [29]. Under the current flow management regime, hydrological connection between the mid-Murray and lower Goulburn rivers is maintained all year, although low flow conditions (< 450 ML per day) often occur during summer in the lower Goulburn River. In the study reach, typical stream width and depth is about 80 m and 3–4 m for the Murray River and 60 m and 2–3 m for the Goulburn River, respectively.

### Fish movement

Golden perch (mean total length [TL] 426 mm, range 315–580 mm, mean weight 1486 g, range 510–3400 g) were collected from the mid-Murray River (3–6 km upstream and downstream of the Goulburn River junction) and lower Goulburn River (3–6 km upstream of the Murray River junction) using a Smith-Root model 5 GPP boat-mounted electrofishing unit (500–1000 volts, 40 Hz, 120 pulses per second) (Table 1). The length of golden perch at maturity is about 200–300 mm for males and 400 mm for females [30]. The sex of fish could not be determined at the time of tagging, but based on length alone most tagged fish are likely capable of spawning (Table 1). Golden perch were tagged from the mid-Murray (*n* = 15) and lower Goulburn (*n* = 12) rivers in April 2007. A further 25 fish were tagged in April 2008 (Murray: *n* = 14, Goulburn: *n* = 11) and 27 were tagged in April 2009 (Murray: *n* = 13, Goulburn: *n* = 14), making a total of 79 tagged fish.

**Table 1:** Details of the tagged golden perch during the study.

River collected	Year	Fish ID	Length (mm)	Weight (g)	River collected	Year	Fish ID	Length (mm)	Weight (g)
Murray	2007	M 01	498	1685	Goulburn	2007	G 01	400	1030
		M 02	390	1000			G 02	415	1270
		M 03	365	800			G 03	460	1700
		M 04	420	1500			G 04	520	2590
		M 05	520	2790			G 05	485	2150
		M 06	490	2400			G 06*^A^*	435	1860
		M 07	410	1100			G 07	505	2480
		M 08	500	2480			G 08	405	1070
		M 09	395	1010			G 09	350	730
		M 10	424	1320			G 10	470	1960
		M 11	497	1975			G 11	495	2430
		M 12	465	1650			G 12	342	700
		M 13	580	3400		2008	G 13	340	1026
		M 14	363	750			G 14	360	610
		M 15	530	2900			G 15	460	2400
	2008	M 16	410	1082			G 16	365	965
		M 17	366	745			G 17	445	2200
		M 18	415	1193			G 18*^A^*	440	1260
		M 19	350	540			G 19	435	1480
		M 20	435	1337			G 20	342	615
		M 21	424	1518			G 21	315	510
		M 22	450	1309			G 22	320	1120
		M 23	430	1582			G 23*^A^*	370	1125
		M 24	470	2270		2009	G 24	370	910
		M 25	510	1963			G 25	420	1310
		M 26	415	1435			G 26	340	590
		M 27	375	711			G 27	500	1858
		M 28	380	806			G 28	430	1504
		M 29	410	1435			G 29	500	2062
	2009	M 30	320	510			G 30	465	2466
		M 31	450	1584			G 31	320	580
		M 32	390	954			G 32	360	610
		M 33	470	1450			G 33	480	2254
		M 34	400	1068			G 34	360	815
		M 35	420	1184			G 35	360	798
		M 36	505	1646			G 36	450	1710
		M 37	505	2280			G 37	470	1845
		M 38	400	860					
		M 39	425	1168					
		M 40	540	2938					
		M 41	520	2668					
		M 42	360	830					
		Mean ± se	438±9	1520±111			Mean±se	413±10	1421±109

Caught and kept by angler (*^A^*)

Fish were transferred from the river into an aerated, 50-L holding container of river water (temperature approximately 15–18°C) and individually anaesthetised (0.03 mL AQUI-S per litre water) (AQUI-S, Lower Hutt, New Zealand). Time to anaesthesia took about 7–8 minutes. Acoustic transmitters (model V13-1L, Vemco, Nova Scotia, Canada; frequency 69-kHz; dimensions: 36 × 13 mm; weight 11 g in air) were implanted into the peritoneal cavity through an incision of about 15 mm, on the ventral surface between the pelvic and anal fins. Estimated transmitter battery life varied depending on year of manufacture (611, 660 and 880 days for fish tagged in April 2007, 2008 and 2009 respectively). Two interrupted external synthetic absorbable monofilament sutures were used to close the incision. Only fish >500 g were tagged to ensure that the transmitter to fish weight ratios remained below ∼ 2% [31]. For external identification, fish were also tagged with an individually coded ‘t-bar’ tag between the second and third dorsal spines. Throughout the procedure the head and gills of fish were immersed in aerated water containing anaesthetic levels of the AQUI-S solution. Each surgery took about 3 minutes. Each fish was placed into a recovery net positioned in the river. Once the fish were observed to maintain their balance and freely swim throughout the holding net (usually after about 10–15 minutes), they were released near their point of capture.

Twelve acoustic listening stations (Model VR2W, Vemco) were deployed in March 2007 in the mid-Murray (*n* = 8) and lower Goulburn (*n* = 4) rivers (Fig. 1). The listening stations were deployed using a length of plastic-coated steel cable attached to logs as anchor points. A float and weight were attached above and below each listening station respectively, to maintain a vertical position. Each listening station was suspended about 1 m above the river bed. The listening stations were set up in pairs to enable movement into or out of different areas (e.g. mainstem, tributary, junction) to be determined. The junction was defined as the area within the Murray River 2 km upstream or downstream of the Goulburn River confluence. *In situ* tests showed that the listening stations had detection ranges of about 100–200 m depending on the physical attributes of the site (e.g. depth, turbulence). Data were downloaded from the listening stations about every three months throughout the study. Acoustic telemetry observations were included only until the estimated transmitter battery life expiry date.

### Timing of spawning

As part of a separate study, drift nets were used to sample golden perch eggs and larvae in the lower Goulburn River and mid-Murray River throughout the spawning seasons of all years from 2003 to 2011 (W.M. Koster, unpubl. data). Data was used from that study to determine whether the movements of tagged golden perch coincided with the timing of spawning. Sampling was conducted at three sites in the lower Goulburn River (Cable Hole, Pyke Road, Yambuna) and one site in the mid-Murray River (Echuca) (Fig. 1) every 2 weeks from September to February in each year. Drift nets were 1.5 m long, with a 0.5-m diameter mouth opening, consisted of 500 µm mesh, and had flow meters fitted to the mouth of the net to measure the volume of water filtered. The nets were set in late afternoon (1600–2000 hours) and retrieved the following morning (0800–1100 hours). Drift samples were inspected in the field to obtain fertilised eggs so that these could be taken to the laboratory for hatching to assist identification. Remaining samples were immersed into a solution of overdosed anaesthetic (4 mL Alfaxan per litre water) (Jurox, Rutherford, Australia) for 10 minutes to euthanase any larvae, and then preserved in 70% ethanol. These samples were sorted in the laboratory under a dissecting microscope, and identified with the aid of a guide [32].

### Data analysis

A Markov transition matrix, with logistic regressions on the diagonals, was used to examine relationships between environmental factors and the probabilities of fish moving between mainstem and tributary locations. The fish had two choices of location: the Murray River or Goulburn River. Markov models deal explicitly with the inherent time (and in this case spatial) dependencies of the data. This is achieved by assuming that the decision about which location to be in next time is affected only by the current location and explanatory variables. The model estimated the probability of maintaining the fish's location in weekly time steps given its current location [33], [34]. The form of the model is given below:
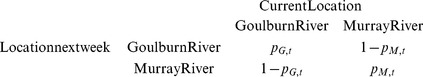



where *p_G,t_* and *p_M,t_* represents the probability that a fish remains at their current location (either the Goulburn River or Murray River respectively) for the next week. Both *p_G,t_* and *p_M,t_* are generalised linear models (GLM) with a binomial distribution using a logit link function.

The explanatory variables examined were selected on the basis that golden perch exhibit strong site fidelity [21]–[23], whilst flow, temperature and spawning season are postulated as likely drivers of movement in the species [22], [24], [25]. Formally the explanatory variables were: (1) where the fish was captured, (2) whether it was spawning season (September–February), (3) mean weekly flow, (4) mean weekly water temperature, (5) percentage change in flow (average flow in the current week compared to average flow in the previous fortnight), (6) the coefficient of variation for the flow in the previous fortnight, (7) percentage change in temperature (average temperature in the current week compared to average temperature in the previous fortnight), and (8) the coefficient of variation for the water temperature in the previous fortnight. Because behaviours may vary according to the spawning season and capture river, interactions between these categorical variables and the other covariates was also considered. To explore all possible effects multi-model inference using all combinations involving spawning season and capture river interacting with each covariate was conducted. Multi-model inference using all models was used to determine the relative importance of each predictor by summing the size corrected Akaike (AICc) weights for each model [35]. Model averages were calculated using all models within 4 of the best model (ΔAICc <4). The validity of the models was assessed using the area under the receiver operating characteristic curve (AUC). The AUC measures the discrimination, that is, the ability of the model to correctly determine whether a fish will remain at their current location, or move rivers. An AUC close to 0.5 suggests poor to minimal discrimination, while close to 1.0 suggests excellent discrimination [36]. The models were constructed using ‘glm’ in the statistical program *R* [37] and the ‘MuMIn’ package for *R* [38] was used for model averaging.

## Results

### Timing, duration and frequency of mainstem–tributary movements

**Figure 2:**
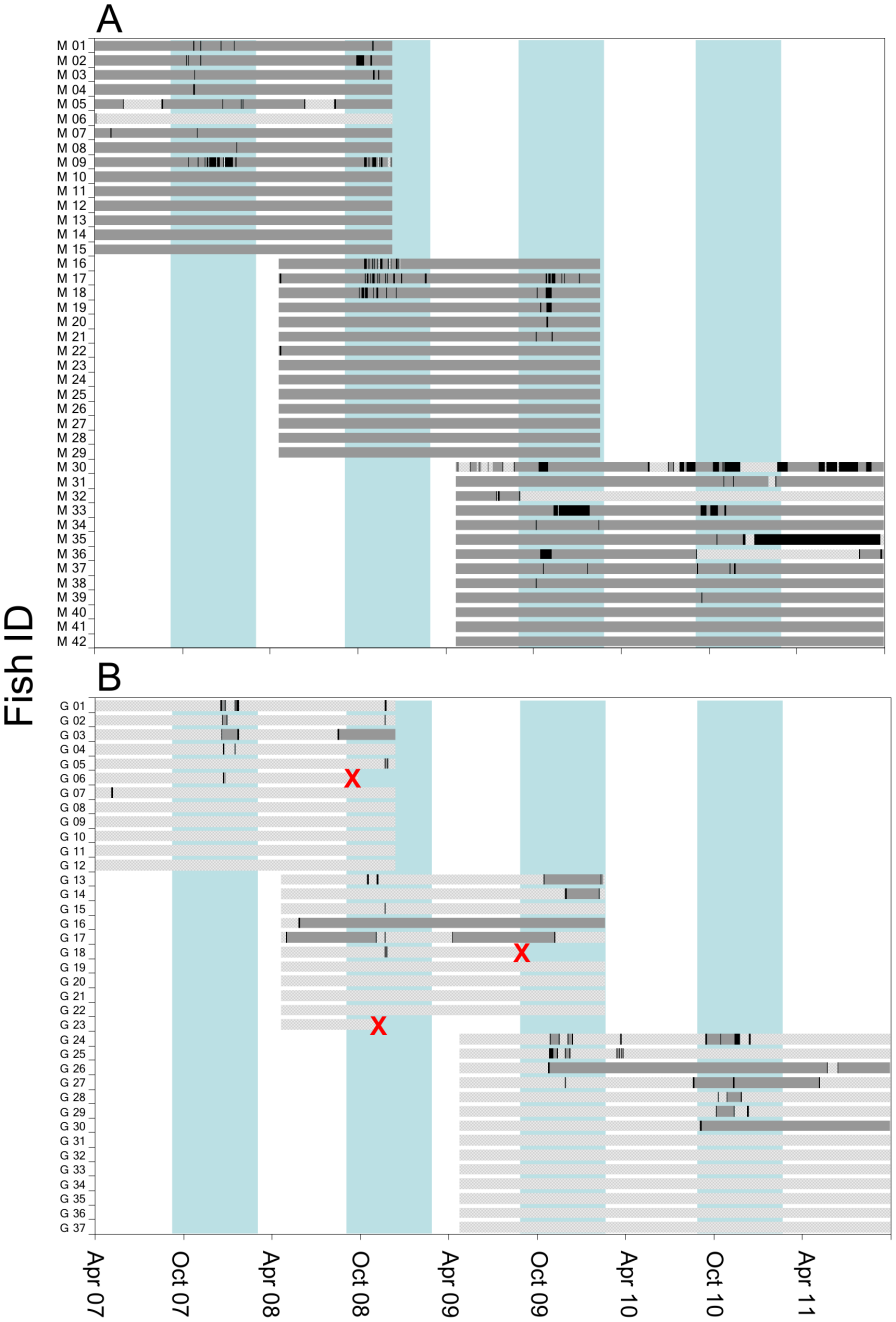
Times during which tagged fish were detected in mainstem, tributary and junction locations. Times (filled bars) during which fish tagged in the Murray River were detected in the Murray River >2 km from junction (dark grey bar), Murray River within 2 km of junction (black bar), and Goulburn River (light grey bar) (*A*), and times during which fish tagged in the Goulburn River were detected in the Goulburn River (light grey bar), Murray River within 2 km of junction (black bar) and Murray River >2 km from junction (dark grey bar) (*B*). Red ‘X’ indicates fish reported as caught and kept by angler. Numbers refer to individual tagged fish. Fish were tagged on three separate occasions (April 2007, April 2008, April 2009). Aqua vertical bar represents spawning season.

**Figure 3:**
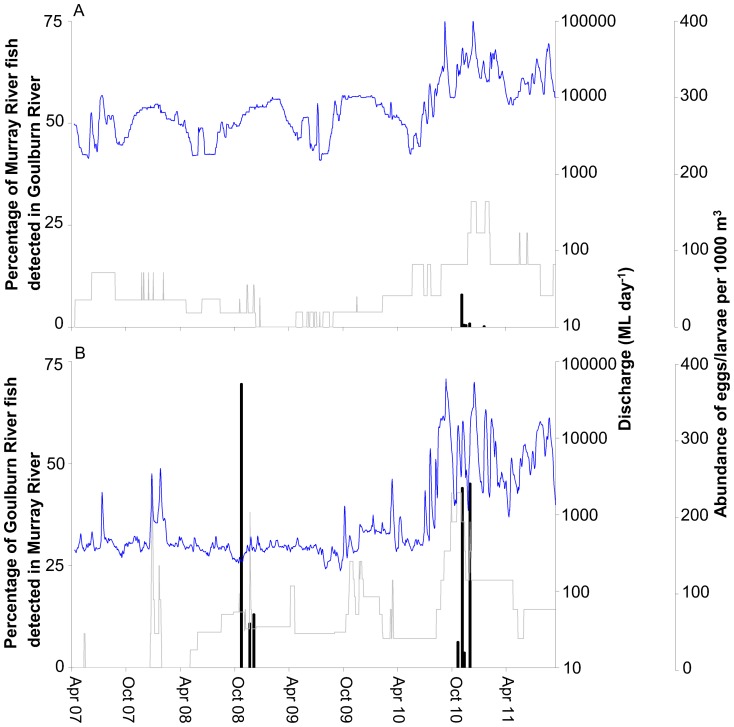
Percentage of mainstem fish detected in tributary and percentage of tributary fish detected in mainstem. Percentage of Murray River fish (grey line) detected in the Goulburn River (*A*) and percentage of Goulburn River fish (grey line) detected in the Murray River (*B*). Blue line represents daily mean discharge in the Murray River at Yarrawonga (*A*) and Goulburn River at McCoys Bridge (*B*). Adjusted total density of golden perch eggs/larvae (black bar) per 1000 m^3^ collected in drift nets in Goulburn River (*A*) and Murray River (*B*).

Of the 79 golden perch tagged during the study, 68 were detected by the listening stations and three were reported by anglers as caught in the Goulburn River and retained (Table 1, Fig. 2). About one quarter (11 out of 42) of fish tagged in the Murray River moved into the Goulburn River (mean TL 431 mm, range 320–520 mm), and just over half (20 out of 37) of fish tagged in the Goulburn River moved into the Murray River (mean TL 433 mm, range 340–520 mm) (Fig. 2, 3). The size range of fish moving between locations was similar to the overall size range of fish tagged (mean TL 426 mm, range 315–580 mm). Most (24 out of 31) individuals that made mainstem–tributary movements returned to the river from which they were originally collected, although seven fish (Murray *n* = 3, Goulburn *n* = 4) did not return to their capture river during the study (Fig. 2, 3).

Movement of Murray River tagged fish into the Goulburn River occurred in all seasons and was not concentrated during any one particular period (Fig. 2, 3). In contrast, movement of Goulburn River tagged fish into the Murray River was most common during late spring and early summer (i.e. October–December) (Fig. 2, 3). The frequency and duration of visits was similar for fish tagged from each river. Of the Murray River fish that entered the Goulburn River, five visited the Goulburn River once and six visited on multiple occasions (Fig. 2). Nine fish from the Goulburn River visited the Murray River once and 11 fish visited on multiple occasions (Fig. 2). The time spent in the Goulburn River per visit by Murray River fish ranged from one day to over 24 months, with most (68%) visits to the Goulburn River lasting <2 weeks. The time spent by Goulburn River tagged fish in the Murray River per visit ranged from one day to 20 months, with most (61%) visits to the Murray River lasting for <3 weeks.

Almost half (48%) of the Murray River fish were detected near the junction with the Goulburn River (i.e. <2 km upstream or downstream) (Fig. 2). Of these fish, two visited the junction once and 18 fish visited on multiple occasions. Most (70%) visits to the junction occurred in spring and summer and most (85%) lasted for short periods (i.e. <1 week). Of the visits by Goulburn River fish to the Murray River, 33% stayed near the junction (i.e. <2 km upstream or downstream), 40% moved a short distance (2–10 km) upstream or downstream into the Murray River, and 27% moved further (> 10 km upstream or downstream) into the Murray River.

### Environmental correlates

**Figure 4:**
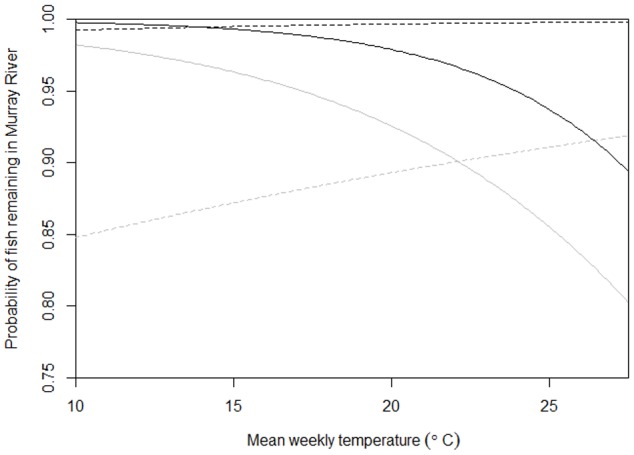
Predicted probability of fish remaining in the Murray River versus mean weekly temperature. Black line represents fish tagged in the Murray River and grey line represents fish tagged in the Goulburn River. Solid line represents spawning season (September–February) and broken line non-spawning season.

For the model examining the probability of movement of fish from the Murray to the Goulburn, the predictors with the greatest relative importance were: where the fish was captured, spawning season, mean weekly temperature, and mean weekly temperature during spawning season (Table 2). If the model is averaged over models with the most evidence (ΔAICc <4), then the non-zero coefficients were related to where the fish was captured, spawning season, and mean weekly temperature during the spawning season (Table 2). Essentially, in any given week fish tagged in the Murray River were highly likely to remain in the Murray River (99% under average conditions): the likelihood of fish tagged in the Goulburn River remaining in the Murray River was 88% less than fish tagged in the Murray River under the same conditions. During the spawning season, the likelihood of a fish in the Murray River staying in its capture river was increased by a factor of more than 17, although this effect was slightly reduced at above-average water temperatures (Table 2, Fig. 4). The AUC for movement of fish in the Murray River was 0.826, suggesting that the discrimination is good.

**Table 2:** Relative importance of predictor variables and parameter estimates for the model averages (models with ΔAICc <4) for the transition models for movement between the Murray and the Goulburn Rivers.

(*A*) Predictor variable	Estimate	Lower CI	Upper CI	Relative Importance
Intercept	1.8232	0.4201	3.2264	
Tagged in Murray River	2.9833	1.8063	4.1603	100.0
Mean weekly flow : tagged in Murray River	0.0000	−0.0001	0.0000	49.0
Mean weekly flow CV : tagged in Murray River	−0.0007	−0.0025	0.0011	23.8
Percentage change in flow : tagged in Murray River	0.7635	−5.0526	6.5797	15.5
Mean weekly temperature : tagged in Murray River	0.0442	−0.0495	0.1379	38.3
Mean weekly temperature : tagged in Murray River : spawning season	−0.1229	−0.3663	0.1205	5.4
Mean weekly temperature CV : tagged in Murray River	−4.0046	−24.3235	16.3144	16.4
Percentage change in temperature : tagged in Murray River	−3.3183	−22.8978	16.2612	21.7
Tagged in Murray River : spawning season	0.1737	−1.3181	1.6655	34.2
Mean weekly flow	0.0000	0.0000	0.0001	85.2
Mean weekly flow : spawning season	0.0000	−0.0001	0.0000	27.9
Mean weekly flow CV	−0.0003	−0.0015	0.0009	77.2
Mean weekly flow CV : spawning season	0.0006	−0.0026	0.0037	21.3
Percentage change in flow	−1.6685	−5.0712	1.7341	57.1
Percentage change in flow : spawning season	3.0913	−2.8064	8.9889	18.0
Mean weekly temperature	0.0405	−0.0409	0.1220	99.6
Mean weekly temperature : spawning season	−0.1897	−0.3020	−0.0774	96.9
Mean weekly temperature CV	−10.0179	−22.0643	2.0284	50.1
Mean weekly temperature CV : spawning season	11.3274	−12.7419	35.3968	19.4
Percentage change in temperature	9.4279	−5.2439	24.0996	65.8
Percentage change in temperature : spawning season	−12.0243	−35.0409	10.9923	30.8
Spawning season	2.8650	0.6558	5.0742	99.9

(*B*) Predictor variable	Estimate	Lower CI	Upper CI	Relative Importance
(Intercept)	4.5165	3.4205	5.6126	
Mean weekly flow	0	−0.0001	0.0001	93.9%
Mean weekly flow : tagged in Murray	0.0001	−0.0001	0.0002	40.5%
Mean weekly flow : tagged in Murray : spawning season	0.0001	−0.0001	0.0004	8.3%
Mean weekly flow : spawning season	0.0001	0	0.0003	79.2%
Mean weekly flow CV	−0.0014	−0.0061	0.0033	76.7%
Mean weekly flow CV : tagged in Murray	0.003	−0.0017	0.0076	37.3%
Mean weekly flow CV : tagged in Murray : spawning season	0.0021	−0.0075	0.0117	3.2%
Mean weekly flow CV : spawning season	−0.0036	−0.0105	0.0033	30.9%
Percentage change in flow	−1.7602	−3.0609	−0.4595	97.9%
Percentage change in flow : tagged in Murray	1.2484	−0.7168	3.2137	45.4%
Percentage change in flow : tagged in Murray : spawning season	2.1932	−2.9271	7.3135	4.9%
Percentage change in flow : spawning season	0.9884	−1.3058	3.2825	41.6%
Mean weekly temperature	−0.0182	−0.0765	0.0401	74.1%
Mean weekly temperature : tagged in Murray	0.0108	−0.1054	0.127	29.6%
Mean weekly temperature : tagged in Murray : spawning season	−0.209	−0.4468	0.0288	10.9%
Mean weekly temperature : spawning season	−0.0513	−0.1472	0.0446	40.6%
Mean weekly temperature CV	6.7495	−6.9487	20.4476	61.2%
Mean weekly temperature CV : tagged in Murray	−17.7972	−39.2437	3.6494	33.6%
Mean weekly temperature CV : spawning season	9.2493	−11.8499	30.3485	20.7%
Percentage change in temperature	−4.9888	−21.9896	12.0119	67.3%
Percentage change in temperature : tagged in Murray	−14.1295	−36.6452	8.3863	31.0%
Percentage change in temperature : tagged in Murray : spawning season	−17.9178	−61.4903	25.6546	4.6%
Percentage change in temperature : spawning season	18.9588	−1.5539	39.4715	44.1%
Tagged in Murray	−1.747	−3.2369	−0.2572	100.0%
Tagged in Murray : spawning season	1.3979	−2.3196	5.1153	69.4%
Spawning season	−0.7716	−2.4056	0.8623	99.9%

Note: The model involves two logistic models, movement of fish currently in the Murray River (*A*) or the Goulburn River (*B*). Flow and temperature represents Murray River at Yarrawonga (*A*) and Goulburn River at McCoy's Bridge (*B*). Confidence intervals are at the 95% level. Relative importance is the sum of the weights for all the models that included that term. Only terms included in the model average have been shown.

**Figure 5:**
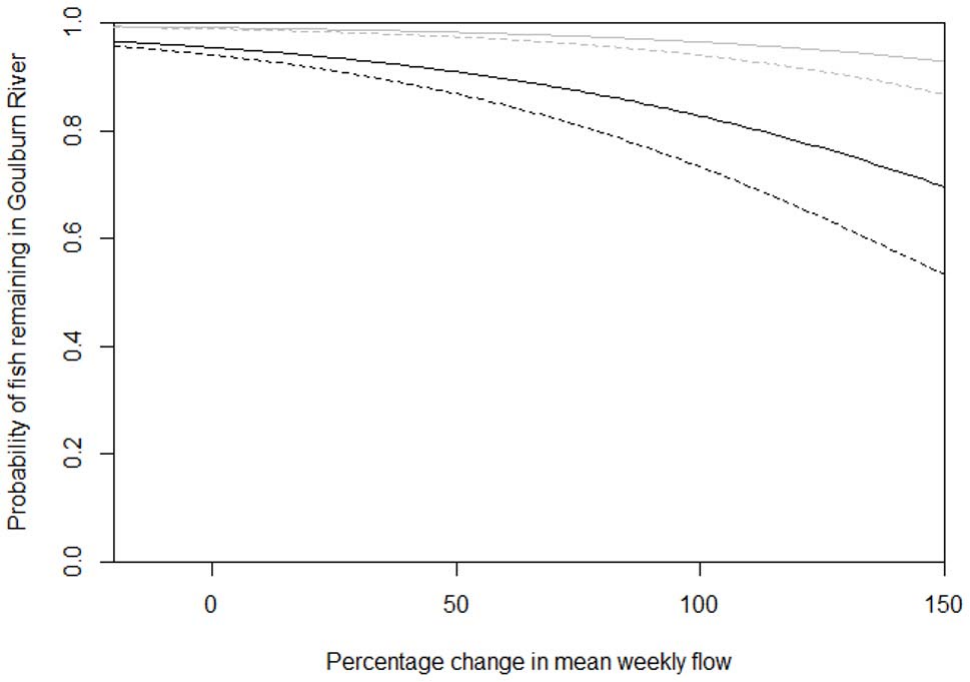
Predicted probability of fish remaining in the Goulburn River versus percentage change in flow. Black line represents fish tagged in the Murray River and grey line represents fish tagged in the Goulburn River. Solid line represents spawning season (September–February) and broken line non-spawning season.

For the model examining the probability of movement of fish from the Goulburn to the Murray, the predictors with the greatest relative importance were: where the fish was captured, percentage change in flow, spawning season, and mean weekly flow (Table 2). If the model is averaged over models with the most evidence (ΔAICc <4), then the non-zero coefficients were related to where the fish was captured, percentage change in flow, and mean weekly flow during the spawning season (Table 2). Essentially, on any given week fish tagged in the Goulburn River were highly likely to remain in the Goulburn River (99% under average conditions): the likelihood of fish tagged in the Murray River remaining in the Goulburn River was 83% less than fish tagged in the Goulburn River under the same conditions. Fish in the Goulburn River were more likely to move into the Murray River during changes in flow in the Goulburn River: a 10% increase in average flow in the current week (when compared to the previous fortnight) reduced the likelihood of fish remaining in the Goulburn River by 16% (Fig. 5). Above average mean weekly flow during the spawning season slightly increased the likelihood of the fish staying in the Goulburn River: during the spawning season, a mean weekly flow of 100 ML above average in the Goulburn River increased the odds of the fish staying by 1%. The AUC for movement of fish in the Goulburn River was 0.778, suggesting that the discrimination is good.

### Spawning

Eggs and/or larvae of golden perch were collected from the mid-Murray River site in November in 2008 and 2010 and were also recorded from other nearby sites in the mid-Murray River in November in 2008, 2009 and 2010 (A.J. King, unpubl. data), coinciding with the timing of movement of fish from the Goulburn River into the mid-Murray River (Fig. 3). Eggs and/or larvae were collected from the Goulburn River only in one spawning season, in November 2010 and January 2011, during a period of major flooding in the lower Goulburn River (Fig. 3).

## Discussion

### Frequency and timing of mainstem–tributary movements

Golden perch moved freely between mainstem and tributary habitats, with 38% of tagged fish undertaking such movements. In most cases, mainstem–tributary movements were characterised by temporary occupation, with fish returning to the river in which they were tagged. However, about 10% of tagged fish did not return to their capture river. This suggests a high level of connectivity between mainstem and tributary populations of golden perch, and corresponds well with the tag-recapture study of Reynolds [24], who found that about 5% of recaptured golden perch had moved from the Murray River into tributaries (e.g. Darling, Murrumbidgee, Wakool). Our findings are also consistent with more recent genetic evidence for high rates of dispersal within drainage basins by golden perch [39].

There were clear differences in the temporal patterns of movement among fish from the two rivers, with movement of tributary fish into the mainstem most common during late spring-early summer, while movement of mainstem fish into the tributary was not concentrated during any one particular period. The movements of tributary fish into the mainstem corresponded with the occurrence of golden perch eggs/larvae in 2008, 2009 and 2010 in the mainstem and it is possible that at least some of the tributary fish moved into the mainstem to spawn. Surveys of drifting eggs and larvae conducted from 2003–2011 (W. M. Koster, unpubl. data) recorded much higher numbers of golden perch eggs and larvae in the mid-Murray River (total = 994, density = 66.0 individuals per 1000 m^3^) than the Goulburn River (total = 143, density = 0.72 individuals per 1000 m^3^), suggesting that the mid-Murray River mainstem is a generally more important spawning location than the Goulburn River tributary. It is possible, however, that the relative importance of the Goulburn and Murray as spawning locations is determined by prevailing hydrological conditions: for example, from 2003–2011 eggs and larvae were only recorded in the Goulburn in three years with 95% of these collected during major flooding that occurred in 2010–11. As previously mentioned, our analyses showed a slight tendency for fish to remain in the Goulburn River during periods of higher than average mean weekly flow. The increased likelihood of Goulburn fish staying in the Goulburn River during spawning seasons with high river flows coincides with increased occurrence of eggs and larvae in the Goulburn River.

In a previous study, O’Connor et al. [22] found that radio-tagged golden perch in the Murray River moved large distances downstream during spring and congregated near the junction of the Murray River and a major tributary (the Wakool River). In the present study, movements of golden perch during the spawning season were not exclusively directed towards the tributary junction at the spatial scale analysed (i.e. within 2 km), with a large (67%) proportion of visits of Goulburn River fish into the Murray River extending 2–10 km or farther (+10 km) into the Murray River. However, most (71%) visits of the Murray River fish to junction occurred during the spawning season. Whilst these findings provide some evidence that areas near the junctions of the mainstem and tributaries may serve as spawning grounds for golden perch, further information on the fine-scale spatial distribution of spawning is required to confirm this suggestion.

The finding that golden perch tend to move out of tributaries into mainstem habitats during the spawning season contrasts with many studies of riverine fishes (particularly salmonids) in the Northern Hemisphere that report upstream migration by adults from the mainstem into tributaries during the spawning season [40]–[42]. Cutthroat trout *Oncorhynchus clarki*, for example, typically use mainstem areas for growth and maturation and move into gravel-rich microhabitats in tributaries to spawn [40]. However, cutthroat trout will spawn in the mainstem if suitable microhabitat is present [41], so their upstream movement into tributaries appears to be driven by the availability of suitable spawning habitat rather than a preference for lower order tributaries *per se*. It is unclear whether golden perch have specific spawning habitat requirements that may be driving the spawning season movement patterns observed here. The only direct observations of spawning by the species have been under controlled aquaculture conditions [43], [44]. Elucidating the spawning habitat requirements of golden perch is therefore an important area for future research on this species.

The irregular timing of movement of mainstem fish into the tributary suggests that such movements are not part of a specific life history event (e.g. reproduction), but could instead represent occasional exploratory behaviour. Although golden perch occupy very restricted home ranges for extended periods outside the spawning season, such periods are punctuated by occasional bursts of more extensive movement (particularly during periods of increased flow) that may be related to the exploration and evaluation of new habitat [21], [23]. Similar findings of home range occupation and occasional extensive movements by golden perch were also reported by O’Connor et al. [22] in the Murray River, and have been reported among various other riverine fishes (e.g. river blackfish *Gadopsis marmoratus,* topminnow *Fundulus notatus*) [45], [46].

### Environmental factors

Mainstem–tributary movements were associated with spawning season, hydrological events, and water temperature. In particular, change in flow in the Goulburn River was associated with increased probability of fish moving into the Murray River, while above-average water temperature (during the spawning season) in the Murray River was associated with increased probability of fish moving into the Goulburn River. These findings support previous suggestions that movement of golden perch is associated with increasing flows and water temperature [22], [24]. The link between movement and flows has important implications for the provision of environmental flows designed to facilitate a frequency and timing of movement between the rivers that mimics the unregulated condition. In particular, it suggests that fish in the Goulburn River respond to variations in flow relative to prevailing conditions, rather than the absolute magnitude of flow. Thus, even under low flow conditions, provided there is sufficient variation in flow or ‘freshes’, the probability of movement might be expected to increase. These findings are consistent with previous studies reporting variation in flow or water level, rather than a particular flow volume, as a trigger for fish movement [15], [47], [48].

In conclusion, the results of this study indicate that a spatially and temporally complex relationship between adult golden perch movement, river discharge and water temperature plays a key role in connecting mainstem and tributary populations of the species. More specifically, the coincident timing of golden perch spawning in the mainstem and movement of tributary fish into the mainstem suggests that reproductive behaviour is a likely driver of the patterns observed. While our study focussed on the movements of adult golden perch, it should be recognised that movements of earlier life history stages also have the potential to facilitate connectivity. Golden perch lay buoyant eggs that drift downstream on river currents [49], [50] and large numbers of juveniles have been observed migrating upstream through fishways in the Murray River [51], [52]. An understanding of the dispersal patterns of all life history stages, from egg to adult, is required to determine with certainty the mechanisms by which connectivity occurs among fish populations in river networks. The patterns of movement we observed have important implications for management and conservation of golden perch, and potentially, other riverine fishes. In particular, the relatively common occurrence of movement across the mainstem–tributary junction suggests that this geographic feature does not function as an impermeable behavioural or demographic boundary between populations in the two rivers. This finding highlights the fact that fish populations do not necessarily conform to artificially constrained management units (e.g. Murray River versus Goulburn River), and demonstrates the need to consider the spatial, behavioural and demographic interdependencies of aquatic fauna across riverscapes [53].

Existing environmental flow recommendations for the mid-Murray River [27] and Goulburn River [26] were developed independently and did not explicitly consider the implications of connectivity for sustaining populations of fish or other aquatic fauna. For example, the exchange of fish between the Goulburn and Murray suggests that populations in the two rivers may serve as reciprocal sources of immigrants and represent an important mechanism to assist the recovery of locally depleted populations following disturbances, such as a blackwater event (i.e. de-oxygenation) in 2010–11 that caused a large-scale fish kill in the lower Goulburn and Murray [54]. Although they are often complex, the behavioural mechanisms and associated environmental conditions that influence connectivity across mainstem–tributary interfaces need to be understood and accounted for during development of conceptual or quantitative models that underpin management actions (e.g. provision of environmental flows) for riverine fishes.
